# One-plate versus two-plate fixation in the treatment of mandibular angle fractures: a retrospective two-centre comparative study

**DOI:** 10.1186/s13005-025-00540-7

**Published:** 2025-09-09

**Authors:** Andreas Sakkas, Mario Scheurer, Robin Kasper, Marcel Ebeling, Alexander Schramm, Frank Wilde, Bernd Lethaus, Johannes Häfner, Rüdiger Zimmerer, Andreas Naros

**Affiliations:** 1 Department of Oral and Maxillofacial Surgery, University Hospital Ulm, Albert-Einstein-Allee 11, 89081 Ulm, Germany; 2 Department of Oral and Plastic Maxillofacial Surgery, Military Hospital Ulm, Ulm, Germany; 3 Department of Oral and Maxillofacial Surgery, University Hospital Tübingen, Tübingen, Germany

**Keywords:** Mandibular angle fractures, Champy principle, Transoral approach, Osteosynthesis, Fixation methods

## Abstract

**Background:**

The treatment of mandibular angle fractures remains controversial, particularly regarding the method of fixation. The primary aim of this study was to compare surgical outcomes following treatment with 1-plate versus 2-plate fixation across two oral and maxillofacial surgery clinics. The secondary aim was to evaluate associations between patient-, trauma-, and procedure-specific factors with postoperative complications and to identify high-risk patients for secondary osteosynthesis.

**Methods:**

In this retrospective two-center cohort study, patients who underwent surgical treatment for mandibular angle fractures via a transoral approach using either 1-plate or 2-plate fixation over a 10-year period were included. Clinic A exclusively performed 1-plate fixation, while clinic B used 2-plate fixation. Demographic, clinical, radiological, and treatment data were analysed. Multivariable analyses were conducted to identify predictors of postoperative complications and secondary osteosynthesis.

**Results:**

A total of 253 patients with 264 mandibular angle fractures were included. Violence was the most common mechanism of injury (*n* = 131; 49.6%). Postoperative complications occurred in 34.6% of the cases at clinic A and 26.8% at clinic B (*p* < 0.0001). Secondary osteosynthesis was required in 9.4% at clinic A and 7.6% of the cases at clinic B, respectively (*p* = 0.6547). Multinomial regression analysis identified smoking, diabetes mellitus, patient noncompliance, left-sided mandibular angle fractures, presence of a third molar (M3), partial M3 eruption, vertical depth A of M3 and horizontal impaction class II (Pell and Gregory) as significant predictors of postoperative complications. Logistic regression analysis identified smoking, diabetes mellitus, patient noncompliance, left mandibular angle fractures, mandibular angle und body fractures and presence of third molars as significant predictors of secondary osteosynthesis. As patient age there is an increased tendency for wound infection and plate/screw loosening (*p* = 0.06). A longer interval between trauma and surgery was associated with a higher risk of postoperative occlusal disturbances (*p* = 0.06). Patients with a longer duration of postoperative IMF were significantly associated with a higher rate of wound infection and secondary osteosynthesis (*p* < 0.05).

**Conclusions:**

Both 1-plate and 2-plate fixation techniques demonstrated acceptable outcomes. Single-plate fixation offers sufficient stability for most mandibular angle fractures with fewer complications, supporting its use in uncomplicated cases. Double-plate fixation may be reserved for complex cases. Higher complication rates were associated with patient-related and anatomical risk factors. Individualized treatment and further prospective studies are needed to refine surgical strategies.

## Introduction

Mandibular angle fractures are among the most common traumatic injuries of the facial skeleton, accounting for 12–42% of all mandibular fractures after condylar fractures [1, 2, 3]. They frequently occur in young male assault victims and are often associated with additional mandibular or dentoalveolar injuries [1, 2, 4, 5, 6, 7, 8, 9, 10, 11, 12, 13].

The management of mandibular angle fractures has evolved significantly over recent decades, particularly regarding fixation techniques [13]. However, due to the anatomical and biomechanical complexity of the mandibular angle — including its thin cross-sectional area, abrupt curvature, masticatory muscle attachments, and the frequent presence of third molars — the optimal treatment approach remains controversial [1, 5, 14]. Consequently, surgical strategies are often influenced by individual case characteristics and surgeon experience [15].

Treatment options range from non-surgical management and closed reduction with intermaxillary fixation (IMF) to open reduction and internal fixation (ORIF) [6, 16, 17]. Although ORIF is generally preferred for displaced fractures, recommendations differ according to fracture type and classification systems such as those of the AO Foundation [1, 17, 18, 19, 20, 21, 22].

The miniplate fixation system, introduced by Michelet and later refined by Champy et al., revolutionized mandibular fracture treatment by proposing the “ideal lines of osteosynthesis” along the superior buccal cortex, targeting the tension zone [5, 23]. This semi-rigid fixation method, championed by Ellis and colleagues and validated by many others [3, 7, 8, 15, 24, 25, 26], enables early function with low complication rates and often eliminates the need for prolonged IMF.

However, some authors argue that single superior miniplate fixation may not provide sufficient functional stability under vertical load, suggesting a risk of distraction at the lower mandibular border [3, 10, 27, 28]. Therefore, it is uncertain whether the patient can withstand occlusal loads due to the changes in the hard and soft tissue components of the masticatory apparatus [10]. In complex or comminuted fractures, the addition of a second miniplate along the inferior mandibular border (compression zone) may enhance stability [29, 30].

In recent years, three-dimensional (3D) miniplates have been introduced to simultaneously stabilize both tension and compression zones, offering biomechanical advantages in selected cases [1, 3, 8, 13, 15, 21, 30, 31, 32].

Despite these advancements, no definitive consensus exists regarding the most reliable fixation method for mandibular angle fractures [15]. While in the past, rigid 2-plate fixation with miniplates and monocortical screws was common, however, this procedure has been associated with a higher complication rate and longer operation time [6, 13, 14, 15, 24, 33, 34, 35]. A systematic review by Wusiman et al. found a significantly lower incidence of wound healing issues, hardware failure, scarring, and paresthesia with 1-plate fixation system [36]. Studies comparing 1-plate and 2-plate fixation are challenged by variability in fracture patterns, patient compliance, and surgeon technique.

To address these gaps, we conducted a retrospective two-center study comparing surgical outcomes following 1-plate versus 2-plate fixation of mandibular angle fractures. The primary objective was to assess postoperative complication rates and the need for secondary osteosynthesis between the two fixation methods. A secondary objective was to investigate patient-, trauma-, and procedure-specific factors associated with postoperative outcomes, aiming to identify high-risk patients who may benefit from tailored treatment strategies. A tailored, evidence-based approach — balancing clinical expertise with patient-centered factors — could help achieve optimal outcomes in the management of mandibular angle fractures.

## Methods

### Patient cohort

This retrospective two-center observational study included all patients with unilateral or bilateral mandibular angle fractures treated via a transoral approach using either 1-plate or 2-plate fixation at two oral and maxillofacial surgery departments. The study period ranged from December 2008 to September 2024 for Clinic A and from May 2014 to September 2024 for Clinic B. Patient records were retrieved from electronic hospital databases.

#### Ethical approval

was obtained from the Ethics Committee of the University of Ulm, Germany and University of Tübingen, Germany. The study is based on a retrospective analysis of anonymized, routinely collected clinical data. As such, the data were already available in institutional records prior to the study initiation, and no interventions or additional data collection outside routine care were performed. The study was conducted in accordance with the Declaration of Helsinki (1964 and its later amendments) and the Medical Research Involving Human Subjects Act (WMO).

Inclusion criteria were: [1] patients of all ages; [2] radiologically confirmed unilateral or bilateral mandibular angle fractures, with or without concomitant mandibular or dentoalveolar injuries; [3] fracture reduction and osteosynthesis with 1-plate or 2-plate fixation using miniplates exclusively through a transoral approach.

Exclusion criteria included: [1] fracture reduction and osteosynthesis via an extraoral approach; [2] use of reconstruction plates; [3] non-displaced fractures treated conservatively with IMF only; [4] concomitant condylar head and neck fractures; [5] missing preoperative CT imaging or postoperative orthopantomograms (OPGs); [6] incomplete medical records.

### Surgical approach

At both centers, patients were treated according to standardized departmental protocols using an intraoral approach. Preoperatively, all patients received a single intravenous dose of ampicillin-sulbactam (Unacid^®^, Pfizer Pharma GmbH) and 250 mg of prednisolone (Solu-Decortin^®^, Merck Serono GmbH).

In most of the cases, four 1.0 × 8 mm IMF screws were placed in the premolar region after disinfection, while in others, a Schuchardt splint was fixed to the upper and lower dentition instead of IMF screws. Access to the fracture site was achieved via a standard vestibular incision extended superiorly along the ascending ramus, followed by subperiosteal dissection and masseter-pterygoid detachment. The fracture was exposed, reduced, and stabilized with intermaxillary fixation (IMF) using wire ligatures.

Third molars (M3) obstructing fracture reduction were surgically removed.

### Protocol - Clinic A (Group A): 1-Plate fixation

**Fig. 1:**
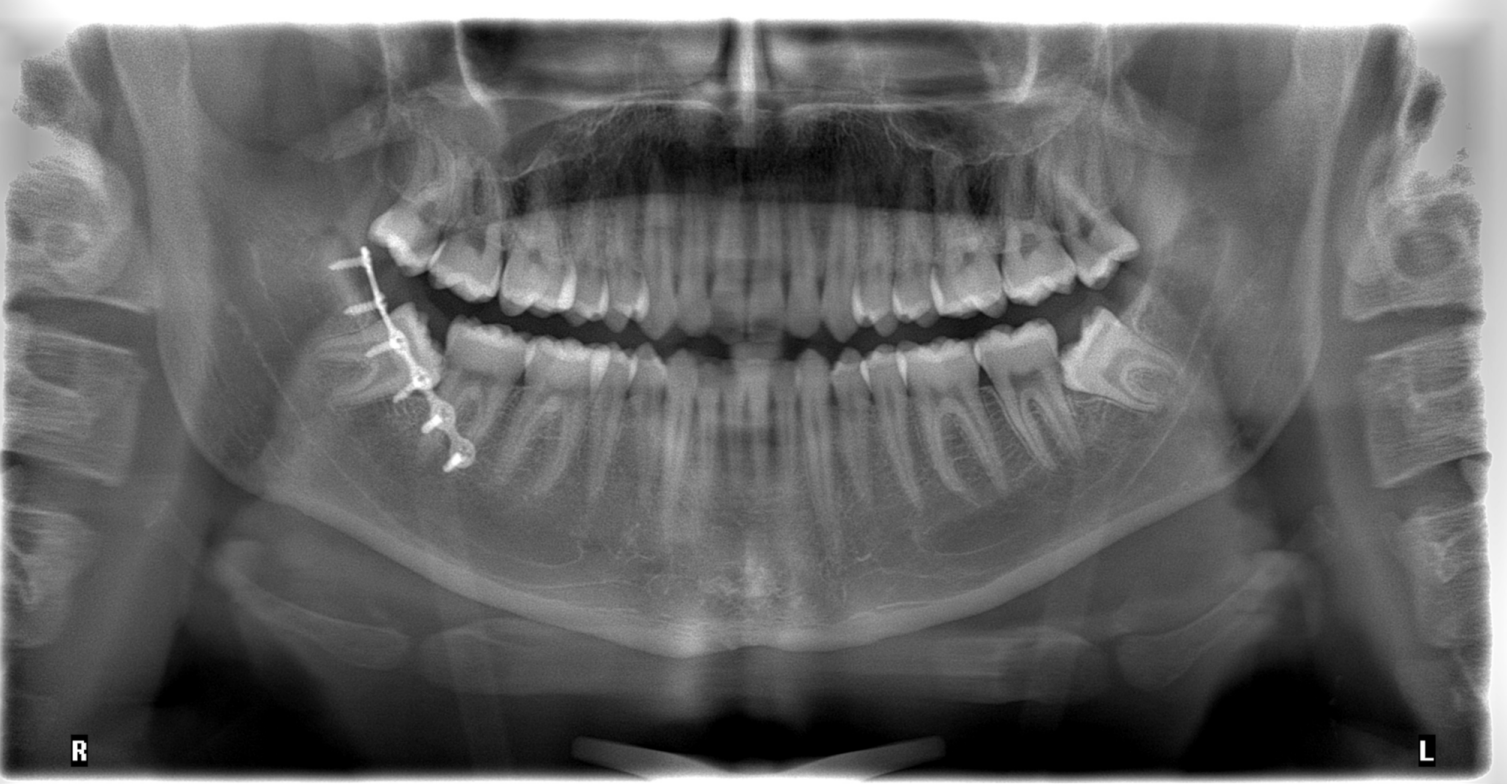
Postoperative orthopantomogram after repositioning of a right mandibular angle fracture and 1-plate fixation

Osteosynthesis was performed with a 4-, 5-, or 6-hole miniplate and monocortical screws (5–9 mm in length, 2 mm diameter) (MatrixMANDIBLE™ Plating System, DePuy Synthes; or KLS Martin Group^®^, Tuttlingen, Germany), placed along the external oblique ridge using a straight manual screwdriver (Fig. 1).

Following fixation, IMF was removed, occlusion was verified, and wound closure was achieved with single Vicryl 3 − 0 sutures (Ethicon, Belgium).

### Protocol - Clinic B (Group B): 2-Plate fixation

**Fig. 2:**
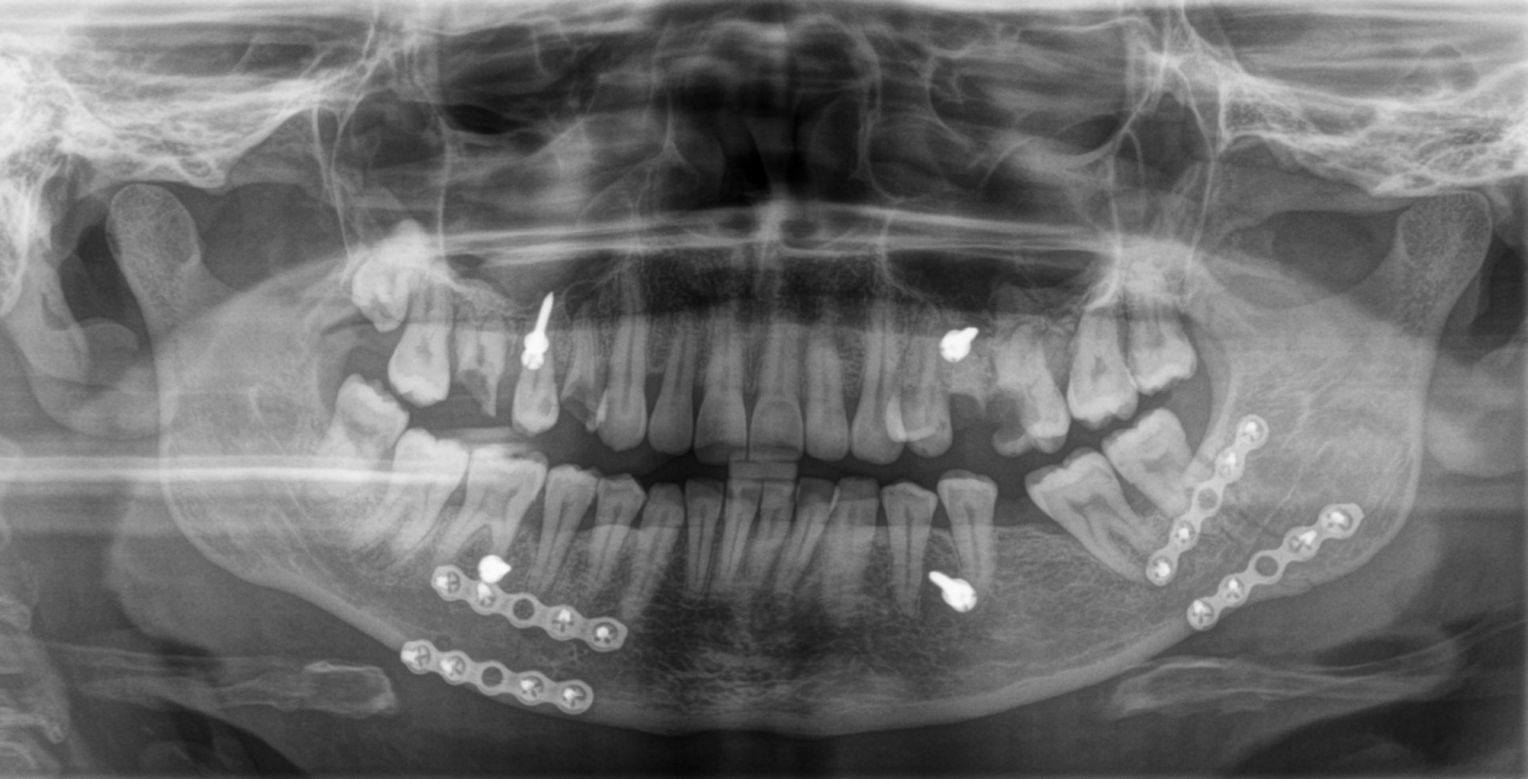
Postoperative orthopantomogram after repositioning of a left mandibular angle fracture and 2-plate fixation

Superior and inferior miniplates (MatrixMANDIBLE™ Plating System, DePuy Synthes) with monocortical screws (5–6 mm in length, 2 mm diameter) were placed: the superior plate along the tension zone (external oblique line or lateral surface) and the inferior plate above the mandibular lower border (compression zone), using a 90°-angled screwdriver (Fig. 2).

In most of the cases, an intraoperative cone beam computed tomography (CBCT) scan (Cios Spine, Siemens Healthineers) was performed to verify fracture reduction, plate positioning, and IMF screw placement. If malreduction was detected, further reduction maneuvers were undertaken under sterile conditions.

After satisfactory fixation and occlusion verification, the IMF was removed, and wound closure was completed with continuous Vicryl 4 − 0 sutures.

### Postoperative care and Follow-up

In Clinic A, an orthopantomogram (OPG) was routinely obtained on the first postoperative day to assess fracture reduction. In Clinic B, where intraoperative CBCT was frequently conducted, a postoperative OPG was not routinely obtained, as the CBCT was considered adequate for postoperative evaluation.

All patients were instructed to maintain a soft diet for four weeks. Postoperative IMF with elastics was maintained for one to three weeks, followed by functional jaw exercises after IMF removal.

Clinical evaluations included wound healing assessment, occlusal relationship in maximal intercuspidation, and assessment of mandibular nerve function immediately postoperatively and during follow-up appointments, scheduled according to clinical need.

### Data collection

Patient data were pseudonymized prior to analysis and included: age at injury; gender; smoking status; medical comorbidities (e.g., diabetes mellitus, cardiovascular disease, immunosuppression); trauma etiology; fracture pattern; clinical symptoms (e.g., occlusal disturbance, inferior alveolar nerve function); presence, position, and morphology of third molars at the fracture site; time between trauma and surgery; intraoperative details (e.g., M3 management, surgical duration, IMF duration postoperatively); and treatment outcomes (complications, secondary osteosynthesis).

Radiological data regarding fracture characteristics and dental status were collected from CT scans and OPGs.

### Study variables

Independent variables included patient-, trauma-, and procedure-specific factors.

Potential predictor variables included:

- Demographic: Age (years).
- Anamnestic: Etiology, smoking status, diabetes mellitus, cardiovascular disease, immunosuppression, patient noncompliance.
- Anatomical: Fracture side, fracture pattern (angle, angle + symphysis, angle + body, bilateral angle).
- Third molar (M3): Presence, position (erupted, extracted, impacted, partially impacted), root morphology (fused, separated, single, unclear), angulation (Winter’s classification), vertical depth (A/B/C), horizontal position (Pell and Gregory classification) [38, 39].
- Surgical: Surgeon level (resident/consultant), M3 management (retained/removed).
- Treatment: Time between trauma and surgery, postoperative IMF duration.

### Outcome measures

- Primary outcome: Postoperative complications (osteomyelitis, wound infection, wound dehiscence, occlusal disturbances, plate/screw loosening, screw in the mandibular canal, late plate exposure). Complications were categorized as minor, when managed conservatively, or major, when a revision of the osteosynthesis in a secondary surgical procedure was required.
- Secondary outcome: Secondary osteosynthesis was defined as full revision surgery involving the removal of the initial osteosynthesis material and placement of new fixation due to postoperative complications (e.g., infection, hardware failure, malocclusion). This definition excludes routine plate removal and minor procedures not requiring re-osteosynthesis.

### Statistical analysis

Data were compiled using Microsoft Excel and analyzed with SAS^®^ version 9.4 (SAS Institute Inc., Cary, NC, USA). Descriptive statistics summarized baseline characteristics. Categorical variables were presented as counts and percentages.

The Kolmogorov–Smirnov test confirmed non-normal distribution for age, trauma-to-surgery interval, and IMF duration; these variables were reported using mean ± SD or range.

Multivariable analyses were conducted to identify associations between predictor variables and postoperative outcomes. Predictor variables included in the regression models were selected a priori based on clinical relevance, biological plausibility, and previously published evidence on factors influencing postoperative outcomes in mandibular fracture management. Variance inflation factors (VIF) were examined, and the highest VIF value observed was 1.272135, indicating no concern regarding multicollinearity. Fisher’s exact test assessed associations between categorical variables and outcomes.

Multinomial regression evaluated relationships between age, trauma-to-surgery interval, IMF duration, and postoperative complications. The key assumptions for this approach include linearity of the logit for continuous predictors, absence of influential outliers in the residuals, and no multicollinearity among predictors. All necessary prerequisites have been thoroughly checked, confirming that this method is appropriate for the research question. Logistic regression evaluated predictors of secondary osteosynthesis.

A *p*-value ≤ 0.05 was considered statistically significant.

## Results

### Demographic distribution

A total of 253 patients with 264 mandibular angle fractures were included in the analysis. The majority were male (226/253; 89.3%), resulting in a male-to-female ratio of 8.37:1. Clinic A included 92 male and 12 female patients, while Clinic B included 134 male and 15 female patients. The mean age at the time of injury was 26.89 ± 9.8 years in Group A (range: 16–52 years) and 30.53 ± 14.94 years in Group B (range: 14–87 years).

### Etiology

Overall, violence was the most common mechanism of injury (*n* = 131; 49.6%). In Clinic A, violence accounted for 42.1% (*n* = 45), followed by pathological fractures after M3 removal (18.7%; *n* = 20) and sports injuries (15.9%; *n* = 17). In Clinic B, violence was the leading cause (54.8%; *n* = 86), followed by pathological fractures after M3 removal (10.2%; *n* = 16) and sports injuries (8.9%; *n* = 14).

### Mandibular fracture patterns

**Fig. 3:**
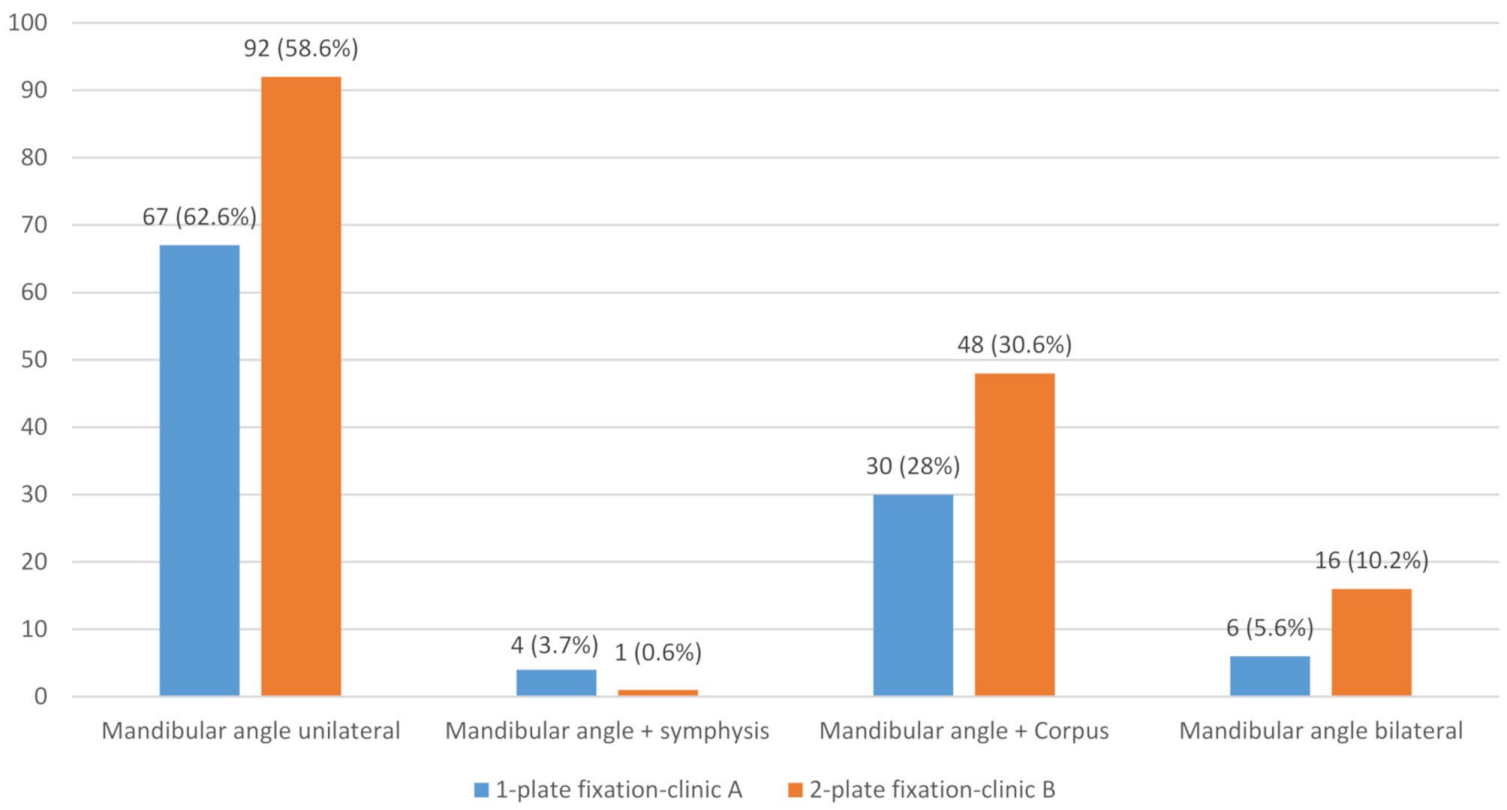
Distribution of mandibular angle fracture patterns in clinics A and B

A total of 264 mandibular angle fractures was recorded in 253 patients. Of these, 159 were unilateral angle fractures. In addition, 5 fractures involved the angle and symphysis, and 78 involved both the angle and mandibular body. Bilateral angle fractures were present in 11 patients, accounting for 22 fractures. All fracture patterns combined yielded a total of 264 mandibular angle fractures. All fracture patterns combined yielded a total of 264 mandibular angle fractures. The distribution of fracture patterns between Clinics A and B is presented in Fig. 3.

### Surgical treatment and Follow-up

In clinic A, the mean interval from trauma to surgery was 2.36 ± 3.52 days (range: 0–19 days). Four-hole osteosynthesis plates were most commonly used (*n* = 49; 45.8%), followed by six-hole plates (*n* = 41; 38.3%), five-hole plates (*n* = 12; 11.2%), and seven-hole plates (*n* = 5; 4.7%). The most frequently used screw length was 7 mm (71.0%; 76/107 cases). Among cases involving a third molar (M3), the M3 was retained in 53 cases (49.5%) and surgically removed in 23 cases (21.5%). Postoperative IMF was applied in 95 cases (88.8%). The mean follow-up period was 238.13 ± 170.2 days.

In clinic B, the mean interval from trauma to surgery was 3.06 ± 3.08 days (range: 0–19 days). For the superior osteosynthesis plate, six-hole plates were most commonly used (*n* = 55; 35.0%), followed by five-hole plates (*n* = 32; 20.4%), seven-hole plates (*n* = 20; 12.7%), and four-hole plates (*n* = 18; 11.5%). The inferior plate most frequently involved five-hole plates (*n* = 42; 26.8%), followed by four-hole plates with an intermediate bar (*n* = 37; 23.6%) and six-hole plates with an intermediate bar (*n* = 34; 21.7%). Screws of 5 mm and 6 mm lengths were predominantly used. Postoperative IMF was applied in 128 cases (81.5%). The mean follow-up period was 255.5 ± 251.6 days.

### Postoperative complications

**Fig. 4:**
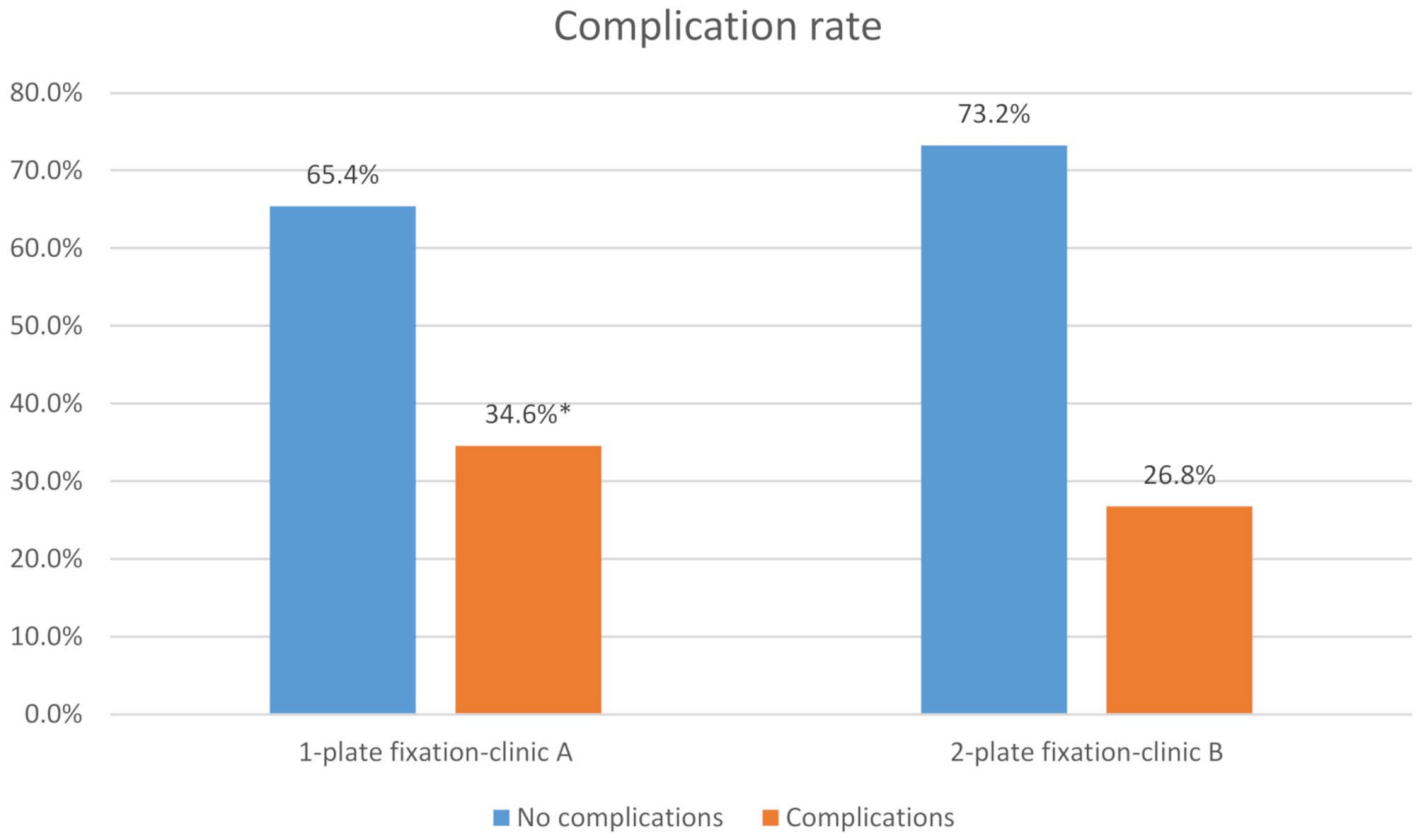
Complication rate related to 1-plate versus 2-plate fixation used *Fisher’s exact test: *p* < 0.0001

**Fig. 5:**
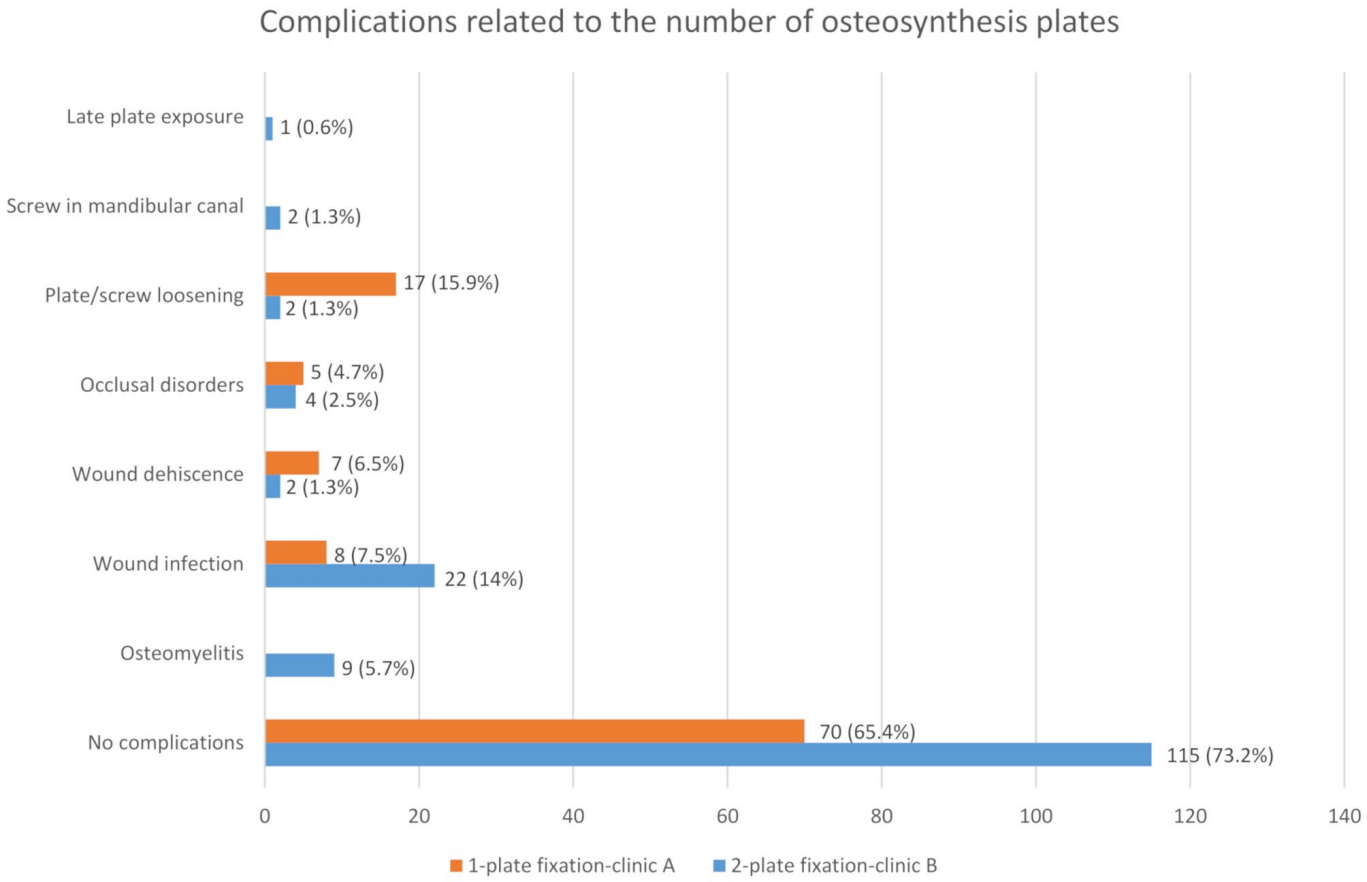
Distribution of specific complications related to 1-plate versus 2-plate fixation used

Postoperative complications occurred in 34.6% of cases at clinic A (*n* = 37) and in 26.8% of cases at clinic B (*n* = 42) during the follow-up period (Fig. 4). This difference was statistically significant (*p* < 0.0001). The distribution of specific complications at both clinics is presented in Fig. 5.

Multinomial regression analysis revealed that smoking, diabetes mellitus, patient noncompliance, left-sided mandibular angle fractures, presence of a third molar, partial M3 eruption, vertical depth A of M3 and horizontal impaction class II (Pell and Gregory) were significantly associated with a higher risk of postoperative complications. These associations are detailed in Table 1.

**Table 1:** Distribution of predictor variables in correlation with postoperative complications

Predictor variables	No complications	Osteomyelitis	Wound infection	Wound dehiscence	Occlusal disorders	Plate/screw loosening	Screw in mandibular canal	Late plate exposure	*p* value*
***Anamnestic variables***									
Smoking									**0.046**
Yes	85	6	18	4	7	8	1	1	
No	100	3	12	5	2	11	1	0	
Diabetes mellitus									**0.0047**
Yes	4	0	0	0	0	2	0	0	
No	181	9	30	9	9	17	2	1	
Cardiovascular disease									**0.1084**
Yes	9	0	4	0	0	1	0	0	
No	176	9	26	9	9	18	2	1	
Immunsuppression									**0.0165**
Yes	1	0	0	1	0	0	0	0	
No	184	9	30	8	9	19	2	1	
Patient noncompliance									**< 0.0001**
Yes	6	4	8	3	0	4	0	1	
No	179	5	22	6	9	15	2	0	
***Anatomy-related variables***									
Fracture side									**0.006**
left	107	5	22	7	7	12	2	1	
right	78	4	8	2	2	7	0	0	
Fracture pattern									**0.07**
Mandibular angle unilateral	109	7	20	5	1	14	2	1	
Mandibular angle + symphysis	3	1	0	0	1	0	0	0	
Mandibular angle + body	56	1	7	4	5	5	0	0	
Mandibular angle bilateral	17	0	3	0	2	0	0	0	
M3 involvement									**0.0315**
present	57	4	14	2	0	8	0	0	
absent	128	5	16	7	9	11	2	1	
M3 location									**0.0205**
erupted	42	3	9	2	0	7	0	0	
extracted	25	0	3	2	0	6	0	0	
impacted	73	1	7	3	6	3	1	1	
partially impacted	13	1	1	1	3	1	1	0	
M3 root configuration									**0.0215**
fused	36	0	7	0	1	0	1	1	
separate	75	5	7	6	7	8	1	0	
single	2	0	2	0	1	3	0	0	
unclear	15	0	1	0	0	0	0	0	
M3 angulation									**0.1239**
buccolingual	3	0	0	0	0	0	0	0	
distoangular	11	1	3	0	1	0	1	0	
mesionagular	64	1	9	1	4	3	1	1	
vertical	49	3	5	4	3	8	0	0	
horizontal	0	0	0	1	1	0	0	0	
germ	1	0	0	0	0	0	0	0	
M3 vertical depth									**0.0305**
A	30	1	3	1	2	7	1	0	
B	26	0	3	3	3	1	0	0	
C	44	1	5	1	4	2	1	1	
M3 horizontal impaction									**0.0025**
I	42	1	3	2	2	8	1	0	
II	36	0	4	3	6	1	1	0	
III	22	1	4	0	1	1	0	1	
***Surgery-related variables***									
Surgeon									**0.0915**
resident	88	3	11	4	4	8	0	0	
consultant	97	6	19	5	5	11	2	1	
Procedure with M3									**0.3013**
remain	110	5	15	3	8	9	2	1	
surgically removed	19	0	2	3	1	2	0	0	

Abbreviations: M3 = third molar; IMF = intermaxillary fixation

•Fisher exact test

As patient age there is an increased tendency for wound infection and plate/screw loosening (*p* = 0.06). A longer interval between trauma and surgery was associated with a higher risk of postoperative occlusal disturbances (*p* = 0.06). Additionally, patients with a longer postoperative IMF duration showed significantly more wound infections. Detailed associations are presented in Table 2.

**Table 2:** Distribution of postoperative complications in correlation with patient age, interval between trauma and surgery and duration of IMF postoperatively

Postoperative complications	Patient age			Interval between trauma and surgery			Duration of IMF postoperatively		
	Odds Ratio	95% CI	*p* value*	Odds Ratio	95% CI	*p* value*	Odds Ratio	95% CI	*p* value*
Osteomyelitis	1.03	0.99–1.08	1.16	1.06	0.88–1.28	0.51	1.07	0.93–1.23	0.33
Wound infection	1.02	1.0–1.05	**0.06**	0.90	0.77–1.06	0.21	1.07	1.01–1.15	**0.03**
Wound dehiscence	1.02	0.97–1.07	0.52	1.00	0.80–1.24	0.98	0.99	0.86–1.14	0.88
Occlusal disturbances	0.96	0.90–1.04	0.33	0.55	0.29–1.02	**0.06**	1.08	0.98–1.19	0.10
Plate/screw loosening	1.03	1.0–1.06	**0.06**	0.80	0.62–1.05	0.10	1.03	0.95–1.12	0.45
Screw in mandibular canal	0.94	0.77–1.14	0.52	0.87	0.46–1.66	0.68	10.7	0.89–2.19	0.48
Late plate exposure	1.05	0.95–1.17	0.33	0.87	0.35–2.17	0.77	10.8	0.85–1.37	0.53

Abbreviations: IMF = intermaxillary fixation; CI = confidence interval

•multinomial regression analysis

In the subgroup of patients with pathological fractures involving bony defects in the third molar (M3) region, 20 cases were recorded in Clinic A (2-plate fixation) and 16 cases in Clinic B (1-plate fixation). In Clinic A, 7 of 20 patients (35.0%) experienced postoperative complications. These included two conservatively managed wound infections and five cases of plate/screw loosening. Among those, two patients (10.0%) required secondary osteosynthesis at 63 and 59 days after the initial procedure. In Clinic B, 3 of 16 patients (18.8%) developed complications. Two patients had wound infections treated conservatively, and one experienced plate/screw loosening requiring secondary osteosynthesis due to pseudoarthrosis 289 days postoperatively (6.3%). Although the overall complication rate was higher in the 2-plate group, the revision rates remained comparable between groups (10.0% vs. 6.3%).

### Secondary osteosynthesis

**Fig. 6:**
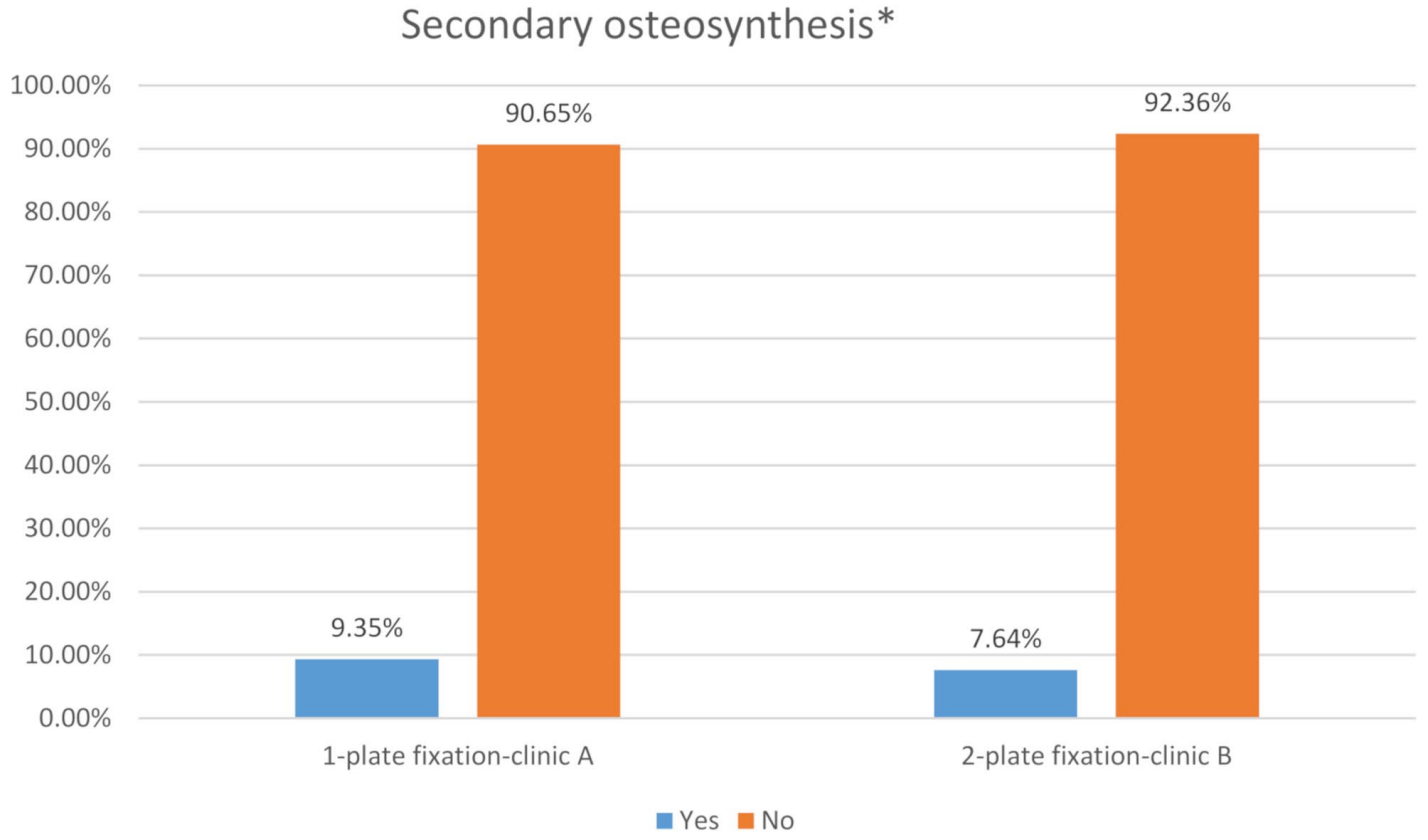
Secondary osteosynthesis related to 1-plate versus 2-plate fixation used *Fisher’s exact test: *p* = 0.6547

Secondary osteosynthesis was required in 9.4% of cases at clinic A (*n* = 10) and 7.6% of cases at clinic B (*n* = 12), with no statistically significant difference (*p* = 0.6547) (Fig. 6).

**Fig. 7:**
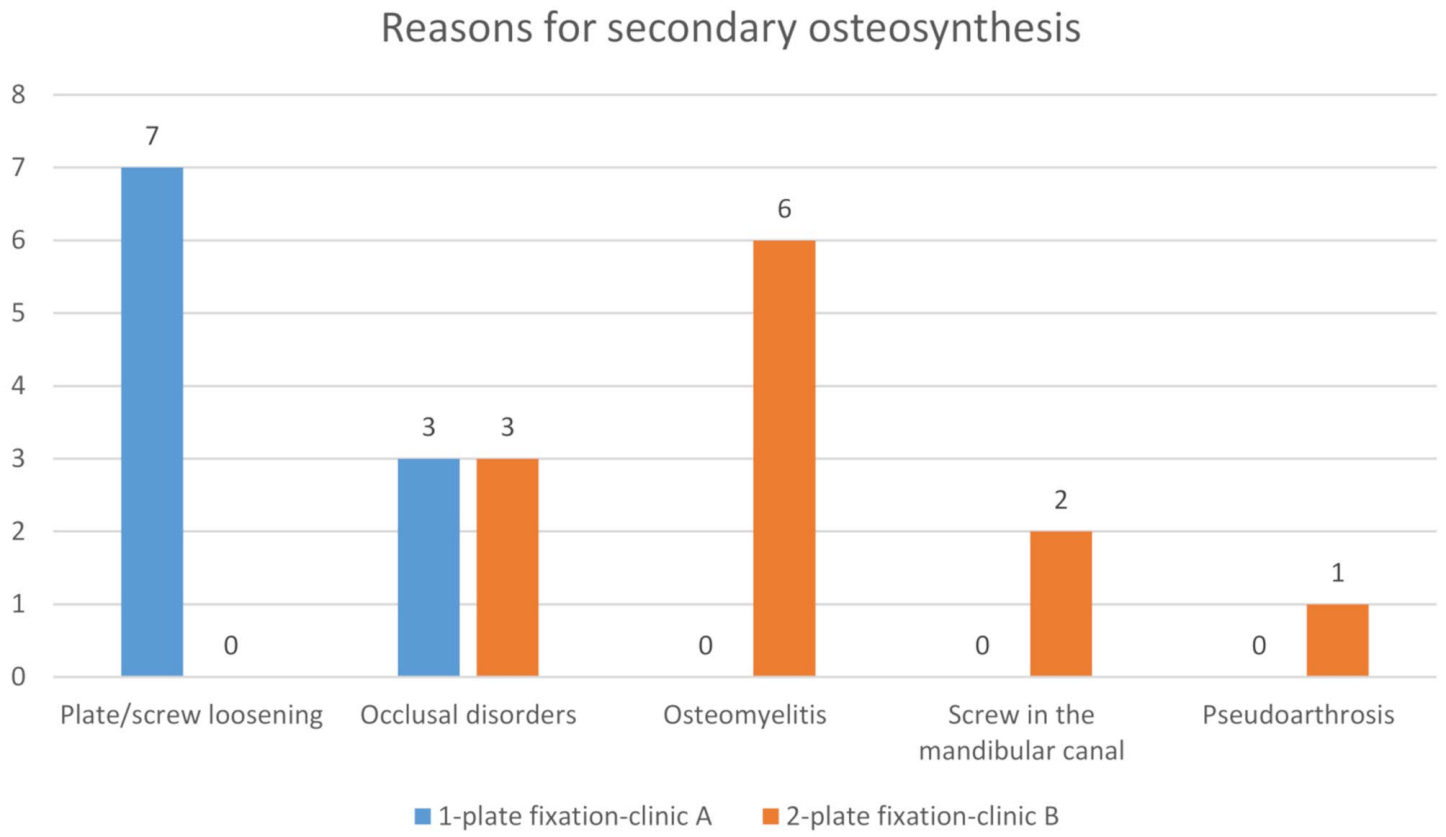
Reasons for secondary osteosynthesis related to 1-plate versus 2-plate fixation

At clinic A, the mean time until secondary osteosynthesis was 28.1 ± 26.1 days (range: 1–63 days). The indications for secondary osteosynthesis included plate/screw loosening (*n* = 7) and occlusal disturbances (*n* = 3), as illustrated in Fig. 7.

At clinic B, the mean time until secondary osteosynthesis was 69.08 ± 91.86 days (range: 1–289 days). Secondary osteosynthesis was performed for osteomyelitis (*n* = 6), occlusal disturbances (*n* = 3), a screw in the mandibular canal (*n* = 2), and pseudoarthrosis (*n* = 1).

Logistic regression analysis identified smoking, diabetes mellitus, patient noncompliance, left mandibular angle fractures, mandibular angle + body fractures and presence of third molars as significant predictors of secondary osteosynthesis. The detailed distribution of predictor variables associated with revision surgery is shown in Table 3.

**Table 3:** Distribution of predictor variables in correlation with secondary osteosynthesis

Predictor variables	Secondary osteosynthesis						Total	*p* value*
	Yes			No				
	*n*	% (of variable group)	% (of secondary osteosynthesis)	*n*	% (of variable group)	% (of secondary osteosynthesis)		
*Anamnestic variables*								
Smoking								**0.0015**
Yes	14	10.77%	63.64%	116	89.23%	47.93%	130	
No	8	5.97%	36.36%	126	94.03%	52.07%	134	
Diabetes mellitus								**0.0065**
Yes	1	16.67%	4.55%	5	83.33%	2.07%	6	
No	21	8.14%	95.45%	237	91.86%	97.93%	258	
Cardiovascular disease								
Yes	0	0.00%	0.00%	14	100.00%	5.79%	14	
No	22	8.80%	100.00%	228	91.20%	94.21%	250	
Immunsuppression								**0.0095**
Yes	0	0.00%	0.00%	2	100.00%	0.83%	2	
No	22	8.40%	100.00%	240	91.60%	99.17%	262	
Patient incompliance								**< 0.0001**
Yes	5	19.23%	22.73%	21	80.77%	8.68%	26	
No	17	7.14%	77.27%	221	92.86%	91.32%	238	
*Anatomy-related variables*								
Fracture side								**0.005**
left	16	9.82%	72.73%	147	90.18%	60.74%	163	
right	6	5.94%	27.27%	95	94.06%	39.26%	101	
Fracture muster								**0.0015**
Mandibular angle unilateral	14	8.81%	63.64%	145	91.19%	59.92%	159	
Mandibular angle + symphysis	2	40.00%	9.09%	3	60.00%	1.24%	5	
Mandibular angle + Corpus	4	5.13%	18.18%	74	94.87%	30.58%	78	
Mandibular angle bilateral	2	9.09%	9.09%	20	90.91%	8.26%	22	
M3								**0.002**
present	18	10.06%	81.82%	161	89.94%	66.53%	179	
absent	4	4.71%	18.18%	81	95.29%	33.47%	85	
M3 position								**0.063**
erupted	6	9.52%	28.57%	57	90.48%	29.38%	63	
extracted	3	8.33%	14.29%	33	91.67%	17.01%	36	
impacted	7	7.37%	33.33%	88	92.63%	45.36%	95	
partially impacted	5	23.81%	23.81%	16	76.19%	8.25%	21	
M3 root configuration								**0.09**
fused	2	4.35%	11.11%	44	95.65%	27.33%	46	
separate	15	13.76%	83.33%	94	86.24%	58.39%	109	
single	1	12.50%	5.56%	7	87.50%	4.35%	8	
unclear	0	0.00%	0.00%	16	100.00%	9.94%	16	
M3 angulation								**0.1389**
buccolingual	0	0.00%	0.00%	3	100.00%	1.86%	3	
distoangular	3	17.65%	16.67%	14	82.35%	8.70%	17	
mesionagular	6	7.14%	33.33%	78	92.86%	48.45%	84	
vertical	8	11.11%	44.44%	64	88.89%	39.75%	72	
horizontal	1	50.00%	5.56%	1	50.00%	0.62%	2	
germ	0	0.00%	0.00%	1	100.00%	0.62%	1	
M3 vertical depth								**0.2024**
A	7	15.56%	46.67%	38	84.44%	30.40%	45	
B	2	5.56%	13.33%	34	94.44%	27.20%	36	
C	6	10.17%	40.00%	53	89.83%	42.40%	59	
M3 horizontal impaction								**0.3353**
I	7	11.86%	46.67%	52	88.14%	41.60%	59	
II	6	11.76%	40.00%	45	88.24%	36.00%	51	
III	2	6.67%	13.33%	28	93.33%	22.40%	30	
*Surgery-related variables*								
Surgery performed due:								**0.008**
resident	10	8.47%	45.45%	108	91.53%	44.63%	118	
consultant	12	8.22%	54.55%	134	91.78%	55.37%	146	
Procedure with M3								**0.2029**
remain	17	11.11%	94.44%	136	88.89%	83.95%	153	
surgically removed	1	3.70%	5.56%	26	96.30%	16.05%	27	

Abbreviations: Abbreviations: M3 = third molar; IMF = intermaxillary fixation, CI = confidence interval

•Fisher exact test

Patients with a longer duration of postoperative IMF were significantly associated with a higher number of secondary osteosynthesis. Neither patient age nor the trauma-to-surgery interval was significantly associated with revision risk. These findings are summarized in Table 4.

**Table 4:** Correlation of predictor variables with secondary osteosynthesis

Predictor variables	Secondary osteosynthesis		
	Odds Ratio	95% CI	*p* value*
Patient age	1.00	0.97–1.03	0.96
Interval between trauma and surgery	0.94	0.80–1.11	0.49
Duration of IMF postoperatively	1.08	1.02–1.15	**0.01**

Abbreviations: CI = confidence interval; IMF = intermaxillary fixation

## Discussion

The management of mandibular angle fractures remains a subject of ongoing debate in maxillofacial surgery, despite substantial advances in osteosynthesis techniques [15, 16]. Our retrospective two-center study compared the outcomes of single-plate (1P) versus double-plate (2P) fixation for mandibular angle fractures, providing further insights into this controversy.

Based on the classical principles established by Champy et al. [23], fixation with a single superior miniplate along the “ideal line of osteosynthesis” has been advocated for decades. This approach aims to neutralize tensile forces at the superior border, allowing compressive forces to be absorbed by bone contact. Champy’s technique, validated by multiple clinical and experimental studies [3, 24], offers a minimally invasive option with sufficient functional stability for simple, non-comminuted fractures [5].

Our results support the continued application of this principle: although the overall complication rate was higher in the 1P group compared to the 2P group (34.6% vs. 26.8%), the rates of secondary osteosynthesis — representing clinically significant failures — were similar (9.4% vs. 7.6%). While the observed difference in overall complication rates between the two groups reached statistical significance, the absolute difference of 7.8% should be interpreted with caution. Given the retrospective design and the relatively modest margin, the clinical relevance of this finding may be limited. Furthermore, the majority of complications in both groups were managed conservatively and did not result in secondary revision of the osteosynthesis. These results suggest that although statistical significance was achieved—likely influenced by the overall sample size—the clinical impact may be less pronounced. As such, treatment decisions should continue to be guided by patient-specific factors and institutional standards, rather than statistical differences alone.

Previous studies provide mixed evidence. Al-Moraissi and Ellis demonstrated in a comprehensive meta-analysis that single miniplate fixation was associated with fewer postoperative complications compared to double miniplate fixation [14]. Our study does not support these findings, as single-plate fixation was associated with a higher complication rate. On contrary, Danda et al. [35] showed in a randomized clinical trial that the addition of a second plate did not confer a significant advantage in reducing infection rates or improving healing outcomes. Ellis and Walker [24], as well as Schierle et al. [29], also reported higher infection rates in patients treated with two miniplates, suggesting that increased surgical dissection and hardware load might compromise soft tissue healing and predispose to infection. Our results support this statement, and the authors believe that the higher wound infection rate after double-plate fixation can be explained by the extended bone exposure, larger sutured wound and longer duration of surgery. However, while the higher rate of wound infection in the two-plate group may suggest an association with greater surgical exposure and potentially longer operating times, we acknowledge that this interpretation should be made with caution. No intraoperative data on surgical duration or extent of dissection were available for analysis. Moreover, surgical time in teaching hospitals is subject to variation due to the involvement of multiple surgeons with differing levels of experience, which may limit its reliability as a surrogate for tissue trauma or complexity.

Regarding revision surgery, osteomyelitis was the most common reason for secondary osteosynthesis after double-plate fixation (50%). These findings caution against the routine use of two plates in fractures otherwise amenable to single-plate fixation. However, the higher rate of plate/screw loosening after single-plate fixation must be highlighted as a potential shortcoming of this fixation. This may be due to the extensive bony defects in the third molar area, particularly in cases of pathological fractures following third molar removal or after intraoperative third molar removal, which can make the osteosynthesis unstable. As a result, 41.1% of the cases with plate/screw loosening after single-plate fixation needed re-osteosynthesis, which was the most common reason for that (70% among the revision cases). The additional use of an inferior osteosynthesis plate in these cases may be recommended, as it could increase stability. Despite the higher complication rate in the one-plate fixation group, the similar revision rates of both fixation methods reported in this study suggest that single-plate fixation remains an effective and reliable treatment option for most cases, especially when considering the shorter surgical time and the reduced personal and financial resources needed [14, 35].

Among patients with pathological fractures involving bony defects in the third molar region, we observed a higher complication rate in the 2-plate group (35.0%) compared to the 1-plate group (18.8%). While plate/screw loosening was more frequent in the 2-plate group, the proportion of cases requiring secondary osteosynthesis remained similar (10.0% vs. 6.3%). These findings do not clearly support the assumption that dual plating improves mechanical stability in compromised bone scenarios. On the contrary, increased hardware may contribute to greater mechanical strain or early loosening. Given the small sample size, these observations should be interpreted with caution, and further prospective studies are needed to explore these trends more conclusively.

Our findings confirm observations made by Steffen et al. [17], who emphasized that deviations from AO principles, as well as patient-related factors such as smoking and systemic disease, significantly contribute to surgical failures requiring secondary interventions. Additionally, we found that patients with a longer duration of postoperative IMF were significantly associated with a higher risk of postoperative complications and secondary osteosynthesis. However, this finding needs to be carefully interpreted. We believe that it is not the postoperative IMF that may lead to a wound infection, but rather a perioperative wound infection, a demanding osteosynthesis or even patient noncompliance may tendent to prolong the duration of postoperative IMF.

Biomechanical studies by Squier et al. [3] confirmed that while superior border fixation offers sufficient load-sharing under controlled conditions, dual plating may improve resistance against vertical forces. However, whether this mechanical advantage translates into superior clinical outcomes remains debatable, especially when considering the increased risk of morbidity associated with more extensive surgical interventions. In this study, there was no significant difference in the rate of postoperative occlusal disorders between the two methods of fixation. However, six out of nine cases with postoperative occlusal disorder at both clinics required revision of the osteosynthesis. Whether the occlusal disorder was detected directly postoperatively or was developed over time remains unknown and needs to be investigated in future studies to determine whether the cause was inadequate intraoperative repositioning or developed from postoperative biomechanical instability.

In addition to standard plating, three-dimensional (3D) miniplates have been developed to simultaneously stabilize both tension and compression zones [5, 8, 15]. Meta-analyses by Wusiman et al. [5, 15] suggested that 3D plates could reduce hardware failure, malunion, and overall complication rates compared to standard two-dimensional plates. Nevertheless, systematic reviews such as that by Kotha et al. [25] emphasized that 3D plates do not consistently outperform single-plate fixation in simple fracture patterns, and their advantages are more pronounced in complex fractures.

The management of third molars (M3) in the fracture line remains another important consideration. In our cohort, the presence of impacted third molars significantly increased the risk of postoperative complications. This observation aligns with findings by Gong et al. [11], who reported that impacted third molars increase the likelihood of unfavourable fracture patterns and delayed healing. In contrast, Sexton et al. [9] suggested that routine removal of third molars may not be necessary unless they directly interfere with fracture reduction or increase infection risk. Other studies, including those by Zanakis et al. [37] and Edouma et al. (50), suggested that retained third molars do not necessarily impair fracture healing, emphasizing the need for individualized decision-making. Rivera-Herrera et al. [38] stressed the importance of thorough radiographic evaluation (e.g., Winter and Pell-Gregory classifications) to better predict potential complications.

Despite ongoing technological advances, including the use of CAD/CAM-designed osteosynthesis plates [30] and finite element analysis (FEA)-validated designs (32, 76), the Cochrane review concluded that no single fixation method has demonstrated superiority across all clinical scenarios [16]. Therefore, a patient-specific, individualized treatment approach remains the gold standard [16, 17], taking into account fracture characteristics, systemic risk factors such as smoking and diabetes, and surgeon expertise (87).

Finally, recent innovations, such as newly designed miniplates introduced by Gamit et al. [10], show promising results in terms of improved bite force restoration. However, widespread adoption of these new technologies requires further validation through large-scale, prospective randomized studies.

A key strength of this study is the relatively large sample size, encompassing 253 patients with 264 mandibular angle fractures treated across two specialized oral and maxillofacial surgery centers over a ten-year period. The strict inclusion and exclusion criteria, consistent intraoral surgical approach, and detailed multivariable analysis of patient-, trauma-, and procedure-specific factors enhance the validity and clinical relevance of our findings. Furthermore, the comparison of two standardized osteosynthesis protocols—single-plate versus double-plate fixation—within distinct institutional frameworks provides valuable real-world insights into current surgical practice.

However, certain limitations must be acknowledged. Firstly, the retrospective design inherently introduces potential selection and information bias. Second, the non-randomized design, in which each participating center adhered to a distinct and exclusive fixation protocol. As a result, potential center-specific differences—such as variations in surgeon expertise, perioperative care, postoperative follow-up durations, and institutional practices—may have influenced the outcomes independently of the fixation technique itself. Therefore, the results should be interpreted as a comparison of institutional treatment protocols, rather than an isolated evaluation of fixation methods. While both approaches were standardized within each center, we acknowledge that this design inherently introduces a risk of center bias. Third, the statistically significant difference in complication rates must be interpreted cautiously, as the absolute difference was small and may not translate into meaningful clinical impact. Also, the lack of intraoperative data such as surgical duration or extent of dissection may prevent definitive conclusions about the cause of higher wound infection rates in the two-plate group. Additionally, in teaching hospital settings, surgical time may be influenced by varying surgeon experience and training and thus may not reliably reflect procedural complexity. Furthermore, some of the observed differences in complication rates may be influenced by institutional protocols rather than the fixation method alone. For example, clinic B routinely performed intraoperative CBCT imaging to verify reduction and fixation and used continuous sutures with finer material (Vicryl 4 − 0), whereas clinic A used postoperative OPGs and interrupted sutures (Vicryl 3 − 0). These differences in perioperative workflow, imaging, and soft tissue management could have contributed to outcome variation and represent potential center-specific confounders beyond the plate configuration. Fourth, since extraoral approaches have been excluded, the indication criteria may have been varied between the two clinical centers. The differences between the centers in using postoperative IMF may also had a contribution to the possible outcomes. Fifth, biomechanical assessments were inferred from existing literature rather than directly evaluated through in vivo analysis or finite element modelling. Since postoperative IMF was applied in most cases at both centers, no conclusion can be drawn about the adequacy of load-sharing between the two fixation methods without postoperative IMF. Sixth, this study did not examine the quality of repositioning in terms of fracture accuracy and occlusal precision, which could also impact our surgical outcomes. Consequently, the study lacks also clinical interpretation of the observed deviations in terms of long-term clinical and functional outcomes. Prospective research with 1-year follow-up assessment is needed to correlate the repositioning results with long-term clinical and functional outcomes such as malocclusion, maximum interincisal opening, TMJ mobility/dysfunction, or masticatory performance. Seventh, some significant predictors of secondary osteosynthesis, such as diabetes mellitus, were based on very small subgroups and may lack statistical robustness. Thus, the study may be underpowered to detect reliable associations for infrequent conditions such as diabetes and these findings should be interpreted with caution. Furthermore, while multivariate analysis adjusted for known confounders, unmeasured variables may still impact postoperative outcomes.

Future prospective randomized controlled trials and standardized biomechanical studies are warranted to validate and expand upon these findings.

### Prospectives

- The increased rate of plate/screw loosening after single-plate fixation may be attributed to insufficient stability in cases with substantial bony defects in the third molar region—particularly in pathological fractures or following intraoperative third molar removal. In such scenarios, the addition of an inferior plate may enhance mechanical support and reduce the need for secondary osteosynthesis. However, although the overall complication rate was higher in the one-plate group, the rate of revision surgery was comparable, suggesting that the presumed biomechanical advantage of dual plating in M3-related defects remains unproven based on our data.
- Conversely, the higher incidence of wound infection and osteomyelitis following double-plate fixation suggests that increased hardware and soft tissue manipulation may elevate the risk of postoperative complications. While extended antibiotic therapy and short-term drainage might reduce this risk, such measures must be critically weighed against current clinical guidelines advocating perioperative prophylaxis only.

## Conclusion

In this retrospective two-center study, we compared the surgical outcomes of single-plate versus double-plate fixation in the management of mandibular angle fractures. Our findings reinforce the validity of the Champy principle, demonstrating that single superior miniplate fixation offers sufficient stability for most uncomplicated fractures, with a comparable rate of secondary revision surgeries to that of double-plate fixation. Although the overall complication rate was slightly higher in the single-plate group, the clinical relevance remained limited, underlining the efficiency and reliability of this less invasive approach.

Patient-related factors such as smoking, diabetes mellitus, and noncompliance, as well as anatomical variables like the presence of impacted third molars or concomitant mandibular body fractures, were significantly associated with increased postoperative complications and the need for re-osteosynthesis. These findings highlight the importance of individualized treatment planning, taking into account not only the fracture characteristics but also the patient’s specific risk profile. However, findings on predictors of secondary osteosynthesis based on very small subgroups may lack statistical robustness and should be interpreted with caution.

While two-plate fixation may offer biomechanical advantages, particularly in complex fracture patterns with bony defect at the third molar area, high functional demands, or cases with patient-specific risk factors for impaired healing, their routine use should be carefully weighed against the potential for increased surgical trauma and postoperative morbidity.

The incoming impact of template-guided patient-specific osteosynthesis plates in the primary endoscopic-assisted repair of subcondylar fractures may also become a game changer in the intraoral treatment of mandibular body/angle fractures.

Future research, particularly prospective randomized studies and investigations into new fixation technologies, is essential to further refine treatment strategies. Postoperative protocols should prioritize early mobilization, strict follow-up, and patient education to ensure optimal functional recovery and reduce long-term complications.

## Data Availability

The datasets used and/or analysed during the current study are available from the corresponding author on reasonable request.

## Declarations

### Presentation

Results of this study will be presented as an oral presentation at the 4. national congress of Oral and Maxillofacial Surgery, 30.10.-01.11.2025, Berlin, Germany.
